# Evaluating ^90^Sr contamination in seafood and human exposure risks near Zhejiang nuclear power plants, China

**DOI:** 10.3389/fpubh.2025.1635278

**Published:** 2025-07-24

**Authors:** Lei Zhou, Rui Wang, Hua Zou, Hong Ren, Peng Wang, Shunfei Yu, Zhongjun Lai, Yiyao Cao

**Affiliations:** ^1^Department of Occupational Health and Radiation Protection, Zhejiang Provincial Center for Disease Control and Prevention, Hangzhou, Zhejiang, China; ^2^School of Public Health, Suzhou Medical College, Soochow University, Suzhou, China

**Keywords:** ^90^Sr, seafood, nuclear power plant, Zhejiang Province, risk assessment

## Abstract

**Objective:**

This work aims to assess the impact of three nuclear power plants (NPPs) in Zhejiang Province on the levels of ^90^Sr in surrounding seafood, and to evaluate the associated health risks, providing a scientific basis for operational monitoring and contributing essential baseline data for nuclear emergency preparedness.

**Methods:**

From 2021 to 2023, the specific activities of ^90^Sr in five types of seafood (fish, shrimp, mussels, crabs, and algae) were monitored in the three NPPs, compared with Zhoushan City, which has no NPP, and the annual effective dose (AED) was estimated.

**Results:**

The results show that the mean specific activities of ^90^Sr in fish, shrimp, mussels, crabs, and algae around the NPPs were found to range from 0.09 to 0.76 Bq/kg, 0.03–0.30 Bq/kg, 0.01–0.22 Bq/kg, 0.11–1.04 Bq/kg, and 0.02–0.95 Bq/kg, respectively. According to the *Kruskal-Wallis* test, there was no statistically significant difference between the ^90^Sr specific activities in seafood from the three NPPs and Zhoushan. The AED due to ^90^Sr intake from seafood for residents living around the three NPPs and Zhoushan are much lower than the recommended threshold of 1.0 mSv/y.

**Conclusion:**

The comprehensive monitoring results demonstrated that the ^90^Sr levels were at background levels and remained stable, consistently well below the limits of the relevant food safety standards, and the dose burden on the population was slight, indicating that the operation of the NPPs in Zhejiang Province did not have a significantly impact on the local seafood.

## 1 Introduction

In the wake of the Fukushima Daiichi nuclear power plant (FDNPP) incident, there has been a notable shift in focus toward not only the major radionuclides released into the environment, but also toward some minor yet highly radiotoxic radionuclides (1), including ^90^Sr. Indeed, following the significant discharges from the FDNPP site in 2011, ongoing releases of ^90^Sr to the marine environment in the form of highly radioactive wastewater have been reported (2).

Given its potential for significant harm, ^90^Sr is among the radionuclides most closely monitored in the event of a severe nuclear accident or nuclear release (3). ^90^Sr has a relatively long half-life (T_1/2_ = 28.8 y) (4), high radio-toxicity, and maintains a high degree of mobility and persistence in the environment (5). Consequently, it is considered to be one of the most potentially hazardous and toxic artificial radionuclides in the environment (6–8). Furthermore, ^90^Sr can be taken up by seafood, and eventually enter the human body *via* the food chain (9). Since the chemical property of ^90^Sr is similar to calcium and its affinity for tissues rich in calcium content like bones and teeth, it can persist within the body for over a decade, leading to significant internal radiation-induced damage (10–14).

Zhejiang Province is located in the southern region of the Yangtze River Delta, with the east of the East China Sea. Due to its strategic coastal location, Zhejiang Province hosts three nuclear power plants, namely Qinshan Nuclear Power Plant (Qinshan NPP), Sanmen Nuclear Power Plant (Sanmen NPP) and San’ao Nuclear Power Plant (San’ao NPP). It is worth noting that the Qinshan NPP is the first nuclear power station independently researched, designed, and constructed by China (15). Sanmen NPP represents the world’s first AP1000 passive safety nuclear power unit (16). San’ao NPP employs China’s self-developed third-generation nuclear power technology “Hualong One” (HPR1000), which possesses independent intellectual property rights (17). As a province with three NPPs, monitoring their operation is particularly critical.

On the other hand, given that Zhejiang Province is home to China’s largest fishing ground, Zhoushan Fishing Ground, thereby contributing to the national seafood supply chain, seafood has become an integral part of the diet of the majority of the population in Zhejiang Province, providing the population with high-quality proteins and essential amino acids (18). The seafood consumption of Zhejiang residents accounts for a considerable proportion in China’s per capita seafood consumption (19). However, ^90^Sr can enter the body through ingestion of seafood by the population, resulting in internal exposure. Therefore, the radioactivity monitoring of ^90^Sr in seafood around nuclear power plants in Zhejiang Province is of great significance. This effort will help determine whether the operation of these NPPs has caused radioactive contamination in coastal marine organisms and whether the consumption of such seafood poses health risks to local residents.

It is notable that there have been few investigations into the specific activity of ^90^Sr in seafood around nuclear power plants in Zhejiang Province. This is due to the considerable technical challenge presented by the necessity for chemical processing and separation in order to determine the levels of pure β-emitters (7). Meanwhile, existing literature reports typically focus on monitoring the specific activity of ^90^Sr in a single type of seafood, rather than conducting systematic testing across multiple seafood categories (20). In this study, we presented the specific activities of ^90^Sr in various seafood species surrounding three NPPs as well as an area of non-nuclear facilities in Zhejiang Province from 2021 to 2023. Based on the time-series observation data, the annual effective dose (AED) was estimated in order to clarify the changing law of seafood radioactivity levels in Zhejiang Province and ensure the safety and security of the food supply. Furthermore, this work provides both a scientific basis for operational monitoring and critical baseline data for nuclear emergency preparedness.

## 2 Materials and methods

### 2.1 Sample collection

**Figure 1:**
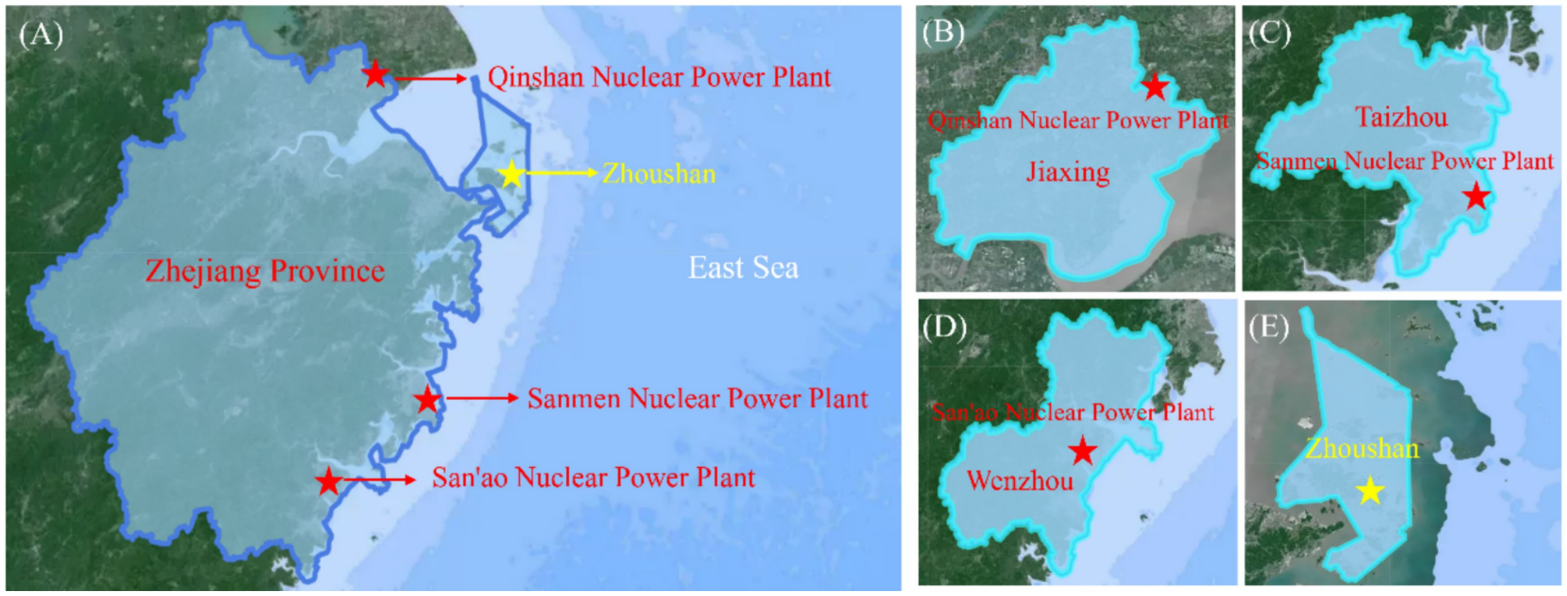
**(A)** Distribution of monitoring sites in Zhejiang Province and the location of cities of **(B)**, **(B)** Qinshan Nuclear Power Plant, **(C)** Sanmen Nuclear Power Plant, **(D)** San’ao Nuclear Power Plant, and **(E)** Zhoushan, the control site.

In this work, fish, shrimp, shellfish, crab and algae were selected around nuclear power plants in Zhejiang Province in 2021–2023. Moreover, to facilitate a more accurate comparison of ^90^Sr activity levels, the city of Zhoushan City was selected as the control point for seafood monitoring. This city does not possess a nuclear power plant and has a high consumption of seafood. Each of the five types of seafood around the NPP and in Zhoushan was collected once a year and used for ^90^Sr detection. The relevant regions and detailed sampling information of seafood are shown in Figure 1 and Table 1.

**Table 1:** Information of sample monitoring point.

City	Nuclear power plant	Seafood	Scientific name	Location		Number of samples per year		
						2021	2022	2023
Jiaxing	Qinshan Nuclear power plant	Mullet	*Mugil cephalus*	120°54′55″E	30°24′57″N	1	1	1
		Prawns	*Penaeus orientalis*	120°56′34″E	30°30′46.97″N	1	1	1
		Razor clam	*Sinonovacula constricta*	120°57′52″E	30°27′46″N	1	1	1
		Portunid	*Portunus trituberculatus*	120°55′52″E	30°24′59″N	1	1	1
		Seaweed	*Saccharina japonica*	120°96′42″E	30°46′23″N	1	1	1
Taizhou	Sanmen Nuclear power plant	Mullet	*Mugil cephalus*	121°5′2.4″E	29 °35′47.04″N	1	1	1
		Eagle-clawed shrimp	*Trachypenaeus curvirostris*	121°35′47.19″E	29 °5′2.52″N	1	1	1
		Oysters	*Ostreidae*	121°35′47.58″E	29 °5′1.82″N	1	1	1
		Blue crab	*Callinectes sapidus*	121°6′34.56″E	29 °34′54.12″N	1	1	1
		Nori	*Porphyra*	121°35′28.32″E	29 °05′12.84″N	1	1	1
Wenzhou	San’ao Nuclear power plant	Eel	*Anguilla*	121°9′1.44″E	27°49′6.97″N	1	1	1
		Prawns	*Penaeus orientalis*	121°2′36.42″E	27°57′5.09″N	1	1	1
		Oysters	*Ostreidae*	120°28′18.70″E	27°9′53.55” N	1	1	1
		Portunid	*Portunus trituberculatus*	121°8′58.24″E	27°49′5.79″N	1	1	1
		Seaweed	*Saccharina japonica*	120°29′38.29″E	27°9′53.24″N	1	1	1
Zhoushan	/	Redhead fish	*Lepidotrigla*	122°12′28.73″E	29°42′8.54″N	1	1	1
		Red shrimp	*Solenocera crassicornis*	122°16′1.23″E	29°52′16.03″N	1	1	1
		Mussel	*Mytilus galloprovincialis*	122°17′58.53″E	29°56′48.63″N	1	1	1
		Portunid	*Portunus trituberculatus*	122°15′13.96″E	29°57′25.75″N	1	1	1
		Seaweed	*Saccharina japonica*	122°27′16.53″E	30°43′39.59″N	1	1	1

### 2.2 Reagents and instruments

The following chemicals were used in the experiment: SrCl_2_·6H_2_O and Y(NO_3_)_3_·6H_2_O (Shanghai Macklin Biochemical Technology Co., Ltd.); Bi(NO_3_)_3_·5H_2_O (Sinopharm Chemical Reagent Co., Ltd.); sodium sulfide, hydrochloric acid, hydrogen peroxide, and nitric acid (Shanghai Lingfeng Chemical Reagent Co., Ltd.), absolute ethanol (Anhui Ante Food Co., Ltd.), oxalic acid (Changshu Yonghua Chemical Technology (Jiangsu) Co., Ltd.), ammonia solution (Hangzhou Longshan Fine Chemicals Co., Ltd.), and di-(2-ethylhexyl)phosphoric acid (60–80 mesh, Beijing Research Institute of Chemical Engineering and Metallurgy, CNNC). The water used in this experiment complies with the Grade 2 water standards specified in GB/T 6682-2008, and all reagents used were of analytical grade.

Low background total *α* and total *β* measuring instrument (LB790 ten-way low background *αβ* measuring instrument, Germany Berthold Technologies), graphite hot plate (HTL-400EX, NanoHeat), muffle furnace (DC-B125/13, Beijing Original Technology Co., Ltd.), electronic balance (AE 200, Mettler Toledo Instruments (Shanghai) CO. Ltd.), drying oven (Binder, Binder Environmental Test Equipment (Shanghai) Co., Ltd.), centrifuge (5810, Eppendorf, Germany).

### 2.3 Sample pre-treatment

All seafood was processed without delay following its arrival at the laboratory, having been transported in a frozen state. The procedure involved washing, selecting edible portions, draining and weighing the samples, which were then placed in an oven at 105°C for drying. After drying, they were carbonized and ashed in a muffle furnace to decompose the organics, a process which requires step-segmental warming. The maximum temperature was maintained at 500°C during the ashing of the samples. Once the ashing process was complete, the sample ash was allowed to cool to room temperature and weighed again in order to obtain the weight of the ash sample and the ash-fresh ratio. In this work, none of the ^90^Sr analytical procedures involved composite samples, and all of our experimental subjects were individual specimens.

### 2.4 Procedure for ^90^Sr analysis of samples

For sample preparation, 5–10 g of sample ash was placed in an evaporation dish, followed by 0.5 mL of strontium carrier (50 mg/mL) and 1 mL of yttrium carrier (20 mg/mL). Then 10 mL of HNO_3_ and 6 mL of H_2_O_2_ were added to remove organic matter and impurities, and after evaporation on a graphite hot plate, the samples were transferred to a muffle furnace at 600°C for calcination for 2–3 h. Once the sample had cooled to room temperature, it was leached with 2 mol/L hydrochloric acid solution (40 mL × 2) and centrifuged at 4,000 rpm for 5 min to retain the supernatant. Subsequently, the residue was washed two times with deionized water and the supernatant was mixed with the previous supernatant, which was retained in a beaker. 10 g of oxalic acid was added to the mixture, and the pH of the solution was adjusted to 2.5 with ammonia. The precipitate was collected by filtration, and then transferred to a muffle furnace for calcination at 800°C for 1 h. The product was completely dissolved with 6 mol/L nitric acid, followed by the addition of 1.0 mol/L HNO_3_, bismuth carrier (20 mg/mL) and 0.3 mol/L sodium sulfide solution, the insoluble matter was filtered out, the filtrate was collected, and its pH was adjusted to 1.

The pretreated filtrate was passed through the Di-(2-ethylhexyl) phosphoric acid (HDEHP) column, and washed with 1.0 mol/L hydrochloric acid (30 mL) at a controlled flow rate of 1.5 s/drop, then rinsed with 1.5 mol/L nitric acid (40 mL). Finally, the filtrate was collected into a Teflon beaker with 6 mol/L nitric acid (30 mL) at a flow rate of 3 s/drop.

The saturated oxalic acid solution (5 mL) was added to the eluent, and its pH was adjusted to 2.5 with ammonia. After aging, the eluate was filtered to produce the precipitate. The dried precipitate was taped to the sample tray and determined with the low background *α*-*β* counter, counting 10 cycles of 100 min each.

### 2.5 Calculation of ^90^Sr specific activity

The specific activity of ^90^Sr in seafood was calculated using the following formula:


$$C=\frac{Ns\;M}{60{\mathrm{\eta WY}}_{Y}e^{-\mathrm{\lambda t}}}$$

where *C* is the specific activity of ^90^Sr, Bq/kg; *N_s_* is the net count rate of the sample, min^−1^; *M* is the ash freshness ratio, g/kg; *ŋ* is the instrumental efficiency, %; W is the sample ash, g; *Y_Y_* is the yttrium chemical recovery, %; *λ* is the decay constant of ^90^Y; and *t* is the intermediate moment from the separation of ^90^Sr, ^90^Y to the measurement, h.

### 2.6 Estimation of annual effective dose (AED)

The radioactivity produced by ingestion accumulates in the body and affects the body’s tissues and organs over a long period of time, causing varying degrees of internal radiation damage (21–23). The annual effective dose (AED, mSv/y), as a radiation protection quantity, is considered an effective tool for radiation exposure risk assessment (15). The resident intake of seafood can be calculated on the basis of the frequency and quantity of seafood consumed by the population, allowing for the estimation of AED.

The AED associated with internal exposure through seafood consumption was calculated by the following equation:


$$\mathrm{AED}=I_{\mathrm{Sr}}\times e{\left(g\right)}_{\mathrm{Sr}}$$

where *I*_Sr_ is the respective annual intake of ^90^Sr in seafood (Bq/y), *e(g)*_Sr_ is the age-dependent dose conversion coefficient for ingestion of ^90^Sr (2.8 × 10^−8^ Sv/Bq).

Referring to the relevant literature on the status of annual consumption of seafood by residents of coastal and island areas in Zhejiang Province (24), the annual per capita consumption of fish, shrimp, shells, crabs and seaweeds for the whole population was shown in Table 2.

**Table 2:** Annual consumption of seafood in Zhejiang.

Region		Annual seafood consumption per capita of the population (kg/year, fresh weight)				
		Fish	Shrimp	Shellfish	Crab	Algae
Coastal area	Qinshan NPP	16.392	4.152	0.779	3.054	0.634
	Sanmen NPP					
	San’ao NPP					
Island area	Zhoushan	32.004	2.865	1.092	3.103	0.333

### 2.7 Quality assurance

Prior to utilizing the LB790 ten-channel low background αβ counter to detect the sample, the electroplating source of ^90^Sr–^90^Y with radioactivity intensity of 1.20 × 10^3^ (Number of particles on the surface/2*π*·min) inspection and standard source scale were employed, and the background measurement cycle and measurement time were consistent with the actual detection of the sample. All instruments utilized were qualified within the verification cycle. The ^90^Sr–^90^Y standard solution (9.78 Bq/g of ^90^Sr in 0.1 mol/L nitric acid) was added to the blank sample for pretreatment and detection, and the discrepancy between the detection results and the reference value of the reference material was within 10%. Ten percent of the samples were taken for parallel sample analysis, and the relative standard deviation of the measured values of the parallel samples was within 10%. The analysts are certified to work, regularly participate in on-site quality control, and the experimental data are accurate and reliable.

## 3 Results and discussion

### 3.1 Specific activity of ^90^Sr in different seafood

The specific activities of ^90^Sr in five types of seafood consumed by residents of Zhejiang are presented in Table 3. The results of the three-year monitoring demonstrate that the specific activities of ^90^Sr in the five types of seafood sampled around the NPP have remained stable and have not undergone significant change. The mean specific activities of ^90^Sr in fish, shrimp, shellfish, crabs, and algae in the vicinity of the nuclear power plant were found to range from 0.09 to 0.76 Bq/kg, 0.03–0.30 Bq/kg, 0.01–0.22 Bq/kg, 0.11–1.04 Bq/kg, and 0.02–0.95 Bq/kg, respectively, which were much lower than the national standards for specific activity of ^90^Sr in seafood and close to the results reported by other literature (25–28). It is noteworthy that ^90^Sr specific activities were significantly higher in fish and crab compared to the other three types of seafood, whereas ^90^Sr specific activities in shellfish were at a lower level, due to the removal of the shells of the shellfish samples used in this study, taking into account the osteophilic nature of ^90^Sr and the fact that it accumulates in the shells of bivalves, together with calcium (29). In comparison to other seafood products, crabs possess a relatively low muscle content. Consequently, during the processing of crabs, the shells were retained, while the inedible parts were removed. However, the main component of crab shells is chitin which bonds calcium strongly (20). Due to the similar chemical properties of strontium and calcium, strontium may be bound to crab shells, leading to the higher specific activity of ^90^Sr in crabs. Moreover, ^90^Sr is metabolized slowly in fish tissues; the effective half-life of ^90^Sr in fish is about 500 days and up to 97% of ^90^Sr is present in fish tissues such as bones, scales, and gills (30). On the other hand, fish and crabs are at a higher trophic level in the food chain compared to the other three types of seafood. As a result, fish and crab exhibit higher ^90^Sr specific activity.

**Table 3:** The specific activities of ^90^Sr in various types of seafood.

Sample species	Year	^90^Sr (Bq/kg)		
		Min.	Max.	Average ± SD
Fish	2021	0.12	0.60	0.35 ± 0.23
	2022	0.12	0.76	0.49 ± 0.28
	2023	0.09	0.67	0.37 ± 0.30
Shrimp	2021	0.06	0.10	0.09 ± 0.02
	2022	0.04	0.16	0.10 ± 0.05
	2023	0.03	0.30	0.14 ± 0.11
Shellfish	2021	0.01	0.09	0.05 ± 0.04
	2022	0.02	0.09	0.05 ± 0.03
	2023	0.03	0.22	0.09 ± 0.09
Crab	2021	0.01	1.04	0.47 ± 0.41
	2022	0.14	0.87	0.43 ± 0.32
	2023	0.24	0.51	0.39 ± 0.11
Algae	2021	0.02	0.58	0.39 ± 0.26
	2022	0.02	0.29	0.18 ± 0.12
	2023	0.04	0.95	0.30 ± 0.44

**Figure 2:**
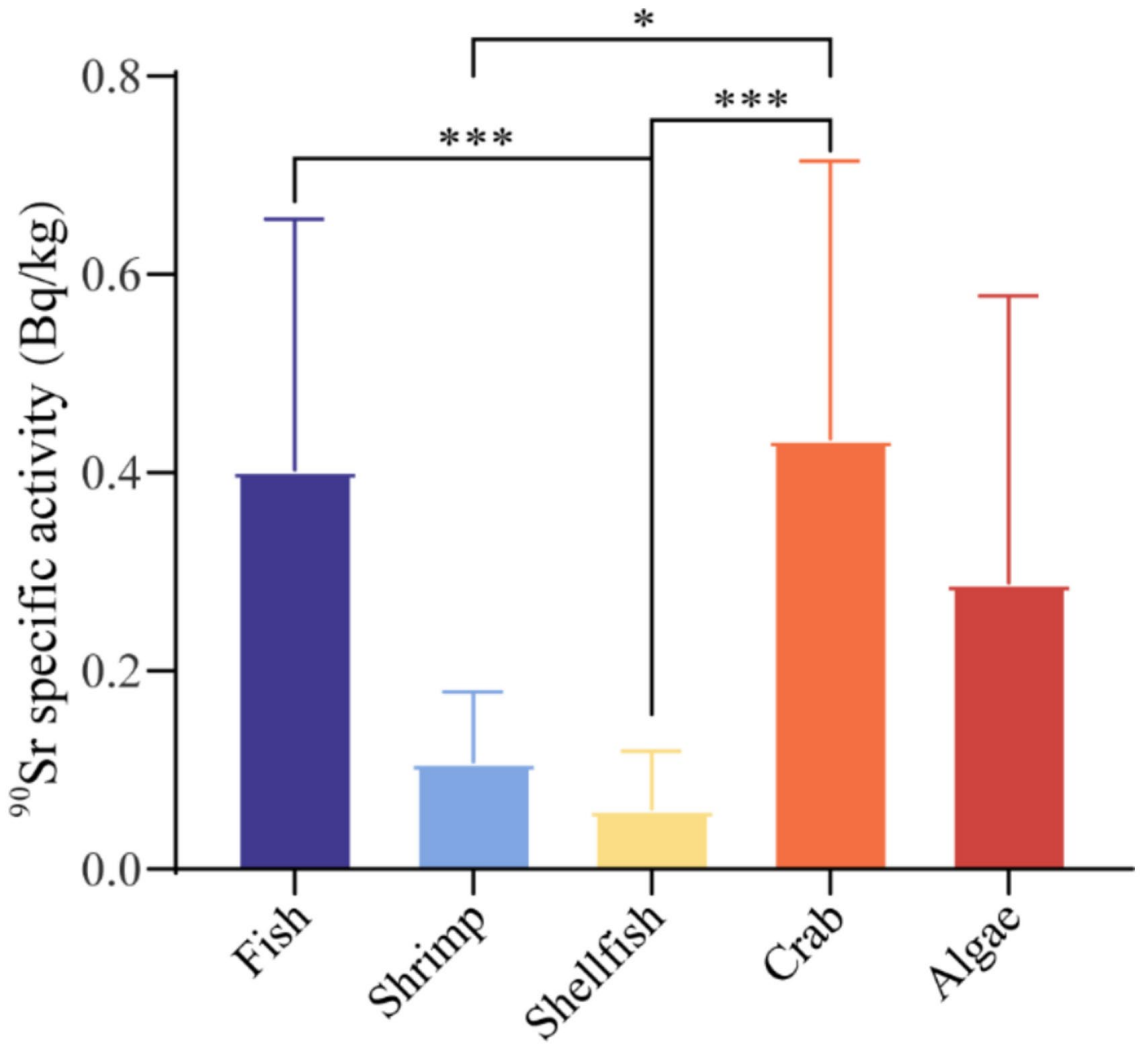
Specific activity of ^90^Sr in different types of seafoods in Zhejiang Province. *p < 0.05, ***p < 0.001.

In this study, the specific activities of ^90^Sr in different types of seafood (fish, shrimp, shellfish, crab, and algae) monitored from 2021 to 2023 were analyzed by the *Kruskal-Wallis* test and the results are presented in Table 4 and Figure 2. The results demonstrated statistically significant differences (*p* < 0.001) in the ^90^Sr radioactivity levels of the five seafood species. A comparison of any two groups of the five seafood products revealed that the *p*-values between fish and shellfish, crab and shellfish, shrimp and crab were all less than 0.05. In particular, the p-values between fish and shellfish, crab and shellfish were all less than 0.001, indicating that there were significant differences in the specific activities of ^90^Sr radioactivity in these groups of seafood. The source of these differences may be due to the different environmental conditions under which the biota grows, as well as to differences in the selectivity and accumulation of ^90^Sr for different types of foodstuffs.

**Table 4:** Comparison of ^90^Sr specific activity in different kinds of seafood in Zhejiang Province from 2021 to 2023.

Sample species	*H*	*p*	SampleX–SampleY	*p*
^a^Fish	28.060	<0.001	a-b	0.052
			a-c	<0.001
^b^Shrimp			a-d	1.000
			a-e	1.000
^c^Shellfish			b-c	1.000
			b-d	0.027
^d^Crabs			b-e	1.000
			c-d	<0.001
^e^Algae			c-e	0.064
			d-e	0.921

### 3.2 Specific activity of ^90^Sr in seafood around different locations

**Figure 3:**
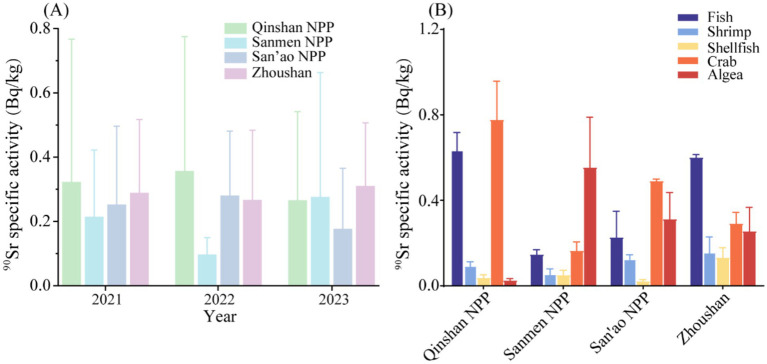
**(A)** Specific activities of ^90^Sr in seafood from various locations in different years; **(B)** Specific activities of ^90^Sr in different types of seafood from different locations.

Seafood is considered to be an important source of protein for people living near nuclear power plants. From the monitoring of seafood from Qinshan NPP, Sanmen NPP, San’ao NPP and the control site Zhoushan, it can be found that the ^90^Sr specific activities of seafood around each nuclear power plant fluctuate normally within the background level and are close to those of the control site Zhoushan from 2021 to 2023, as shown in Figure 3A. A comprehensive investigation was conducted to assess the levels of ^90^Sr in various seafood types at multiple monitoring points (Figure 3B). The findings revealed that the specific activities of ^90^Sr in all seafood categories were markedly below the established limit value, as defined by the national standard. The ^90^Sr specific activity in shrimp and shellfish around Zhoushan is higher than in the other three NPPs. This can be attributed to the unique geographical location of the Zhoushan fisheries at the confluence of the Yangtze River, Yong River and Qiantang River and the estuary of the three rivers (31), has turned the waters of Zhoushan into an important outlet for radionuclides from the Yangtze River, resulting in the accumulation of certain radionuclides in seafood. On the other hand, Zhoushan Fishery is known for its rich biodiversity. The area is shaped by complex interactions of ocean currents and topography, creating a habitat that is favorable for a wide range of marine organisms. It serves as a vital spawning and feeding ground for a multitude of shrimp species (32). It was also observed that there were minor fluctuations in the specific activities, which were attributed to a number of objective factors, including fluctuations in the detection instruments and changes in background levels. However, it was noted that the specific activities remained at the background level and did not exhibit any significant increase.

The statistical analyses (*Kruskal-Wallis* test) of the three NPPs and the control site demonstrated that the specific activities of ^90^Sr in seafood from disparate locations were analogous (Table 5). Furthermore, no significant difference was observed (*p* > 0.05), which provides additional evidence that the three nuclear power plants were operating stably.

**Table 5:** Comparison of specific activities of ^90^Sr in seafood from different locations.

Region	Average specific activity of ^90^Sr in seafood (Bq/kg)	*H*	*p*
Qinshan NPP	0.32 ± 0.36	1.849	0.604
Sanmen NPP	0.20 ± 0.25		
San’ao NPP	0.24 ± 0.20		
Zhoushan	0.29 ± 0.20		

### 3.3 Specific activities of ^90^Sr in seafood from different years

**Figure 4:**
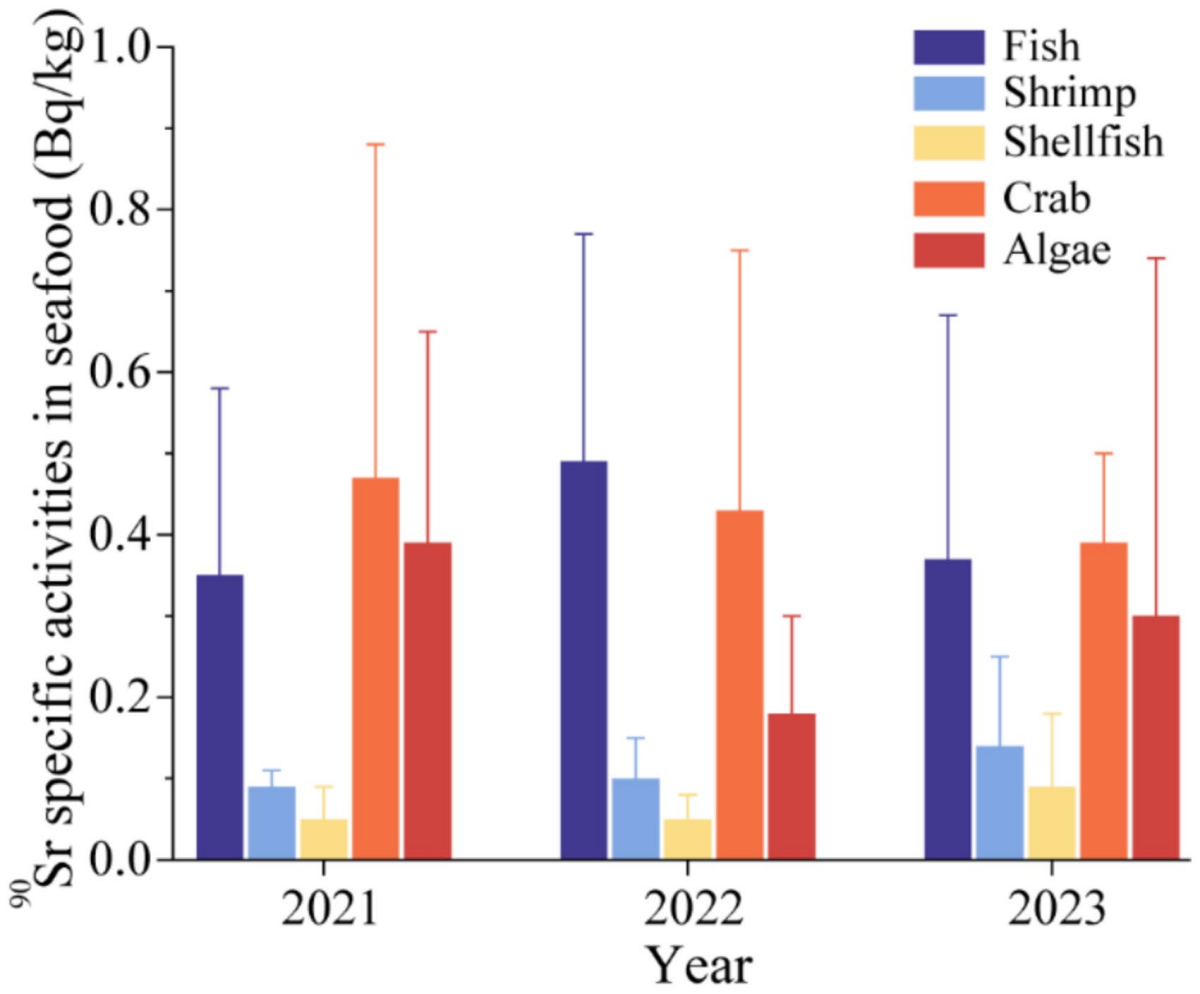
^90^Sr specific activity of seafood in Zhejiang Province in different years.

The specific activities of ^90^Sr in the five types of seafood monitored in different years are presented in Figure 4. The results demonstrate that there was no significant inter-annual variation in the levels of ^90^Sr radioactivity in the five types of seafood during the three-year period, and that the results exhibited a tendency toward stability. The *Kruskal-Wallis* test was carried out to analyze the ^90^Sr specific activities of fish, shrimp, shellfish, crab and algae over the period from 2021 to 2023. The results demonstrated no statistically significant difference (*p* > 0.05), as illustrated in Table 6. This finding indicates that there was no notable alteration in specific activities of ^90^Sr among the five seafood types between 2021 and 2023. Furthermore, the specific activities of ^90^Sr remained stable and remained below the national standard limits. This indicates that the nuclear power plant operated in an effective and environmentally responsible manner, with no discernible adverse effects on marine life.

**Table 6:** Comparison of the trends of ^90^Sr specific activities in five types of seafood in Zhejiang Province between 2021 and 2023.

Sample species	Year	*H*	*p*
Fish	2021	0.589	0.745
	2022		
	2023		
Shrimp	2021	1.435	0.488
	2022		
	2023		
Shellfish	2021	0.355	0.837
	2022		
	2023		
Crab	2021	0.010	0.995
	2022		
	2023		
Algae	2021	0.939	0.625
	2022		
	2023		

Our team has been engaged in a prolonged period of research, with the objective of monitoring the ^90^Sr specific activity in foodstuffs in the vicinity of three nuclear power plants in Zhejiang Province (33–35), as shown in Table 7. Furthermore, the specific activities of ^90^Sr in seafood detected in 2020 at the Ningde and Fuqing nuclear power plants, which are located in close proximity to the latitude and longitude of the three nuclear power plants in Zhejiang Province, ranged from 0.03 to 0.75 Bq/kg and 0.03–0.39 Bq/kg, respectively (22). The specific activities of ^90^Sr in fish around Rooppur Nuclear Power Plant in the People’s Republic of Bangladesh, which is also on the Asian plate, ranged from 0.02 to 1.55 Bq/kg (36). The results of this study for ^90^Sr specific activity in seafood did not exhibit a significant change when compared to the results of the team’s previous monitoring and monitoring results around other nuclear power plants. Furthermore, the ^90^Sr specific activity we observed in seafood were similar to those reported in the global monitoring studies (20, 26, 37, 38), indicating stable operation of nuclear power plants in Zhejiang Province.

**Table 7:** ^90^Sr specific activity of seafood from different location in different years.

Year	Location	Nuclear power plant	Sample	^90^Sr (Bq/kg)	Reference
2012–2019	Jiaxing, China	Qinshan Nuclear Power Plant	Mullet	0.40–1.10	(33)
2011–2020	Taizhou, China	Sanmen Nuclear Power Plant	Mullet	0.21–0.88	(34)
2021–2022	Wenzhou, China	San’ao Nuclear Power Plant	Sea bass	0.12–0.47	(35)
2020	Ningde, China	Ningde Nuclear Power Plant	Seafood	0.03–0.75	(22)
2020	Fuqing, China	Fuqing Nuclear Power Plant	Seafood	0.03–0.39	(22)
2016–2017	Bangladesh	Rooppur Nuclear Power Plant	Fish	0.02–1.55	(36)
1996–2007	Japan	/	Crab	0.03–0.32	(20)
1998–2008	Japan	/	Algae	0.02–0.05	(37)
2015–2017	Korean Peninsula	/	Seafood	0.01–0.02	(26)
2001–2010	Bulgarian Black Sea	/	Macroalgae and mussel	0.23–0.85	(38)
2021–2023	Jiaxing, China	Qinshan Nuclear Power Plant	Seafood	0.02–1.04	This work
2021–2023	Taizhou, China	Sanmen Nuclear Power Plant	Seafood	0.03–0.95	This work
2021–2023	Wenzhou, China	San’ao Nuclear Power Plant	Seafood	0.01–0.55	This work
2021–2023	Zhoushan, China	/	Seafood	0.05–0.62	This work

### 3.4 Dose assessment

**Figure 5:**
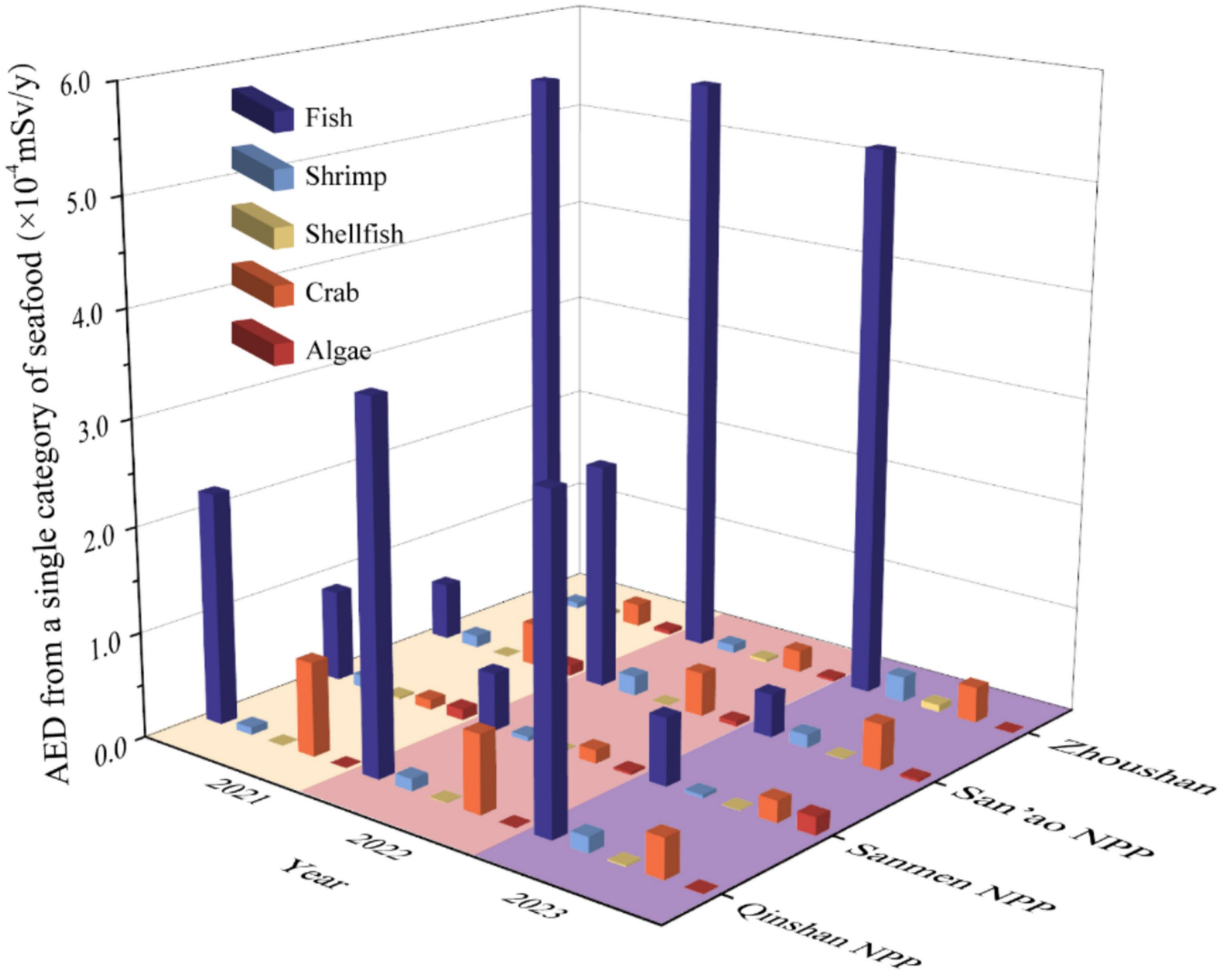
The AED from a single category of seafood in different NPPs and control site Zhoushan from 2021 to 2023.

Based on the survey of relevant literature on the annual consumption status of seafood by residents of coastal cities in Zhejiang Province (24), the AED due to the intake of ^90^Sr from a single type of seafood by the residents around the nuclear power plant and Zhoushan City in 2021–2023 was estimated, as shown in Figure 5. It is noteworthy that among the five categories of seafood, marine fish contributed the largest AED, which may be associated with the dietary habits of residents of Zhejiang Province, where fish has the highest annual per capita consumption. Additionally, this can be attributed to the practical considerations for residents, for whom fish is more readily accessible. From 2021 to 2023, the AED due to individual types of seafood in the vicinity of the three nuclear power stations and Zhoushan remained at a stable level, with no significant fluctuations observed. Furthermore, the AED accumulated from total seafood consumption over the period from 2021 to 2023 was estimated (Table 8). The results demonstrated that the AED due to total seafood at Qinshan Nuclear Power Station, Sanmen Nuclear Power Station, San’ao Nuclear Power Station and Zhoushan were 0.55 × 10^−4^ to 4.36 × 10^−4^ mSv/y, 0.76 × 10^−4^ to 1.20 × 10^−4^ mSv/y, 1.01 × 10^−4^ to 2.81 × 10^−4^ mSv/y, 5.72 × 10^−4^ to 5.85 × 10^−4^ mSv/y, respectively. The AEDs from seafood in Zhoushan City were higher than those in the three nuclear power plant regions of Zhejiang Province, primarily because Zhoushan is an island area, while the Qinshan NPP, Sanmen NPP and San’ao NPP are located in coastal areas—leading to distinct differences in seafood consumption habits. Furthermore, the Zhoushan city encompasses China’s largest fishing ground, Zhoushan Fishery, renowned for its high marine biodiversity and abundant fishery resources. This ecological advantage ensures greater accessibility of diverse seafood for local residents, leading to significantly higher seafood consumption compared to populations near nuclear power plants. Consequently, the AED attributed to seafood in Zhoushan exceeds that in the nuclear power plant vicinity. However, these values are considerably below the acceptable level of 1.0 mSv/y set by the International Commission on Radiological Protection (ICRP) for the general public, indicating that the dose burden on the population is slight and that the operation of the nuclear power stations has no discernible impact on public health. Furthermore, the anthropogenic radionuclide ^137^Cs appears in about 40% of seafood dose assessments due to its radiological significance in routine and accidental releases from nuclear facilities, its background presence due to global fallout and its relative ease of measurement (39). By comparing the AED due to ^137^Cs in seafood reported in the relevant literature (40), we found that the AED due to ^90^Sr in seafood monitored in this work is close to the reported value, which is consistent with the results reported by IEAE on the contribution to annual ingested dose from radionuclides in diet (41).

**Table 8:** AED due to seafood from different nuclear power plants and Zhoushan, 2021–2023.

Location	AED due to seafood (×10^−4^ mSv/y)				Limit value (mSv/y)
	2021	2022	2023	Range	
Qinshan NPP	3.17	4.36	0.55	0.55–4.36	1.0
Sanmen NPP	1.20	0.76	1.06	0.76–1.20	
San’ao NPP	1.18	2.81	1.01	1.01–2.81	
Zhoushan	5.72	5.90	5.85	5.72–5.85	

## 4 Conclusion

The specific activities of ^90^Sr in seafood from three NPPs and the control site Zhoushan were monitored from 2021 to 2023. The results showed that the specific activities of ^90^Sr in seafood was at background levels for the period 2021–2023, which were at the same level as the results of domestic and international studies, and well below the food safety limit. The specific activity of ^90^Sr was found to be significantly higher in fish and crab than in the other three types of seafood. From the monitoring of seafood from three NPPs, it can be found that the ^90^Sr specific activities of seafood around each NPP fluctuate normally within the background level and are close to Zhoushan. There was no significant inter-annual variation in the levels of ^90^Sr radioactivity in the five types of seafood during the three-year period, and that the results exhibited a tendency toward stability and no statistically significant difference (*p* > 0.05). Additionally, the AED values associated with seafood consumption near the three NPPs and in Zhoushan are significantly lower than the acceptable standard of 1.0 mSv/year. This study addresses the scarcity of research on ^90^Sr specific activities in seafood around NPPs. By monitoring the ^90^Sr levels in seafood, we established baseline data that provides a solid foundation for evaluating nuclear power plant operations and safeguards food safety for provincial residents.

## Data Availability

The original contributions presented in the study are included in the article/supplementary material, further inquiries can be directed to the corresponding author.
